# Seismogeodetic P‐wave Amplitude: No Evidence for Strong Determinism

**DOI:** 10.1029/2019GL083624

**Published:** 2019-10-29

**Authors:** D. E. Goldberg, D. Melgar, Y. Bock

**Affiliations:** ^1^ Institute of Geophysics and Planetary Physics, Scripps Institution of Oceanography University of California San Diego San Diego California USA; ^2^ Department of Earth Sciences University of Oregon Eugene Oregon USA

**Keywords:** earthquake determinism, earthquake early warning, seismogeodesy

## Abstract

Whether the final properties of large earthquakes can be inferred from initial observations of rupture (deterministic rupture) is valuable for understanding earthquake source processes and is critical for operational earthquake and tsunami early warning. Initial (P‐wave) characteristics of small to moderate earthquakes scale with magnitude, yet observations of large to great earthquakes saturate, resulting in magnitude underestimation. Whether saturation is inherent to earthquake dynamics or rather is due to unreliable observation of long‐period signals with inertial seismic instrumentation is unclear. Seismogeodetic methods are better suited for broadband observation of large events in the near‐field. In this study, we investigate the deterministic potential of seismogeodetically derived P‐wave amplitude using a dataset of 14 medium‐to‐great earthquakes around Japan. Our results indicate that seismogeodetic P‐wave amplitude is not a reliable predictor of magnitude, opposing the notion of strong determinism in the first few seconds of rupture.

## Key Points

- Seismogeodesy provides a more accurate P‐wave amplitude than high‐pass filtered seismic‐only methods due to its broadband sensitivity.
- Magnitude saturation from P‐wave characteristics is inherent to large earthquake dynamics and not due to instrumental limitations.
- P‐wave amplitude measurements are not predictive of final magnitude for medium‐to‐large magnitude earthquakes.

## 1 Introduction

Earthquake early warning (EEW) systems are designed to provide stakeholders with an estimate of the timing and intensity of shaking following an earthquake in their vicinity. Earthquake magnitude is of first order importance in ground motion prediction, thus, reliable characterization of magnitude in the early seconds of observation is a major target for EEW. The timeliness of source size estimates for EEW is particularly important for large continental earthquakes, where major faults coincide with population centers (e.g., San Andreas Fault System, California, USA) and warning lead times are especially short.

Theories have been proposed to suggest that initial rupture observations can be used to anticipate the final slip amount and rupture extent (i.e., early processes are *deterministic* of final properties). One possibility is that the dynamic stress drop at rupture initiation, or initial rupture surface area, is proportional to the energy flow that advances rupture, and therefore initial rupture observations are indicative of the probability of rupture extent (Zollo et al., 2006). A study of source time function databases shows that distinct peaks precede the maximum moment rate, and that observations of only several peaks can be used to estimate earthquake magnitude (Danré et al., 2019). Another study supplemented by near‐source displacements from high‐rate Global Navigation Satellite Systems (GNSS) observations postulate that after an initiation phase, the ruptures of large magnitude earthquakes organize into a slip pulse whose properties scale with magnitude, allowing very large earthquakes to be distinguished from lesser earthquakes within about 10 s (Melgar & Hayes, 2019).

From an operational standpoint, including early warning, the value of such a process is undeniable; it would allow rapid estimation of final earthquake magnitude, enabling more effective ground motion prediction at those sites closest to the earthquake origin. However, earthquake source time functions are difficult to estimate in real‐time. Instead, different features of observed early onset P‐waves have been studied as possible indicators of final magnitude: initial period (Wu & Kanamori, 2005), maximum predominant period (Nakamura, 1988; Olson & Allen, 2005), displacement amplitude (Noda & Ellsworth, 2017; Wu & Zhao, 2006), and other nucleation phase properties (e.g., Colombelli et al., 2012; Ellsworth & Beroza, 1995). However, observational support for a strongly deterministic mechanism remains controversial, both because of limited datasets and because there is strong evidence of magnitude saturation above magnitude ~7.5 (e.g., Rydelek & Horiuchi, 2006; Trugman et al., 2019). Recent studies of seismic data present evidence that P‐waves initially develop in a statistically indistinguishable manner, regardless of final magnitude, with seismograms only becoming distinguishable with later observation (Meier et al., 2016, 2017; Noda & Ellsworth, 2016). Further complicating the measurement of P‐wave amplitudes at nearby stations is that in the near‐field (i.e. on the order of the rupture length) of large earthquakes, the P and S waves are difficult to separate (Madariaga et al., 2019).

It remains to be clarified whether magnitude saturation above ~M_w_7.5 from initial P‐wave observations is inherent to the physics of large earthquake rupture or rather if saturation is specific to the inertial seismic instruments used in these studies. For rapid magnitude estimation, inertial sensor observations such as strong‐motion accelerometer waveforms are doubly integrated to estimate displacements. However, any inertial seismic instrument will be unable to distinguish between rotational and translational motions. When rotations are present, these are misinterpreted as translations leading to improper recordings of the true translational motion. This problem is only exacerbated for large ground motions typical of great earthquakes (e.g., Trifunac & Todorovska, 2001), where reliable early warning is arguably most important. A number of other potential error sources have been identified that make integration of inertial seismic data inaccurate (e.g., Boore & Bommer, 2005). These small errors, collectively known as baseline offsets, are amplified by integration, introducing an unphysical drift and long‐period biases in the resulting waveforms (e.g., Melgar et al., 2013). As a result, integrated waveforms are commonly high‐pass filtered (e.g., Boore et al., 2002) to produce a physically realistic displacement waveform devoid of drift. Consequently, baseline‐corrected waveforms lack the long period and static offset information potentially required to resolve the true earthquake size leading to the well‐known issue of magnitude saturation most recently seen in operational systems during the 2011 M_w_9.1 Tohoku‐oki earthquake (Hoshiba et al., 2011). The filtering process described is commonplace in P‐wave algorithms used in EEW, with waveforms typically filtered at a corner period of 13 s (e.g., Brown et al., 2011). The resulting frequency content roughly corresponds to that of an M_w_7 earthquake. Since M_w_7 is approximately the size at which magnitude saturation is observed, it is ambiguous whether magnitude saturation is a source effect or an artifact of inertial instrumentation and data processing.

High‐rate GNSS observations have become commonly used to observe the dynamic and permanent displacements resulting from moderate to great earthquakes in real time (e.g., Bock & Melgar, 2016). GNSS instrumentation measures displacement directly in a non‐inertial reference frame, avoiding the integration issues that affect traditional seismic measurements and allowing more accurate representation of ground displacements for even the largest events. Peak ground displacements (PGD) measured with GNSS provide unsaturated magnitude estimates (Crowell et al., 2013; Melgar et al., 2015; Ruhl et al., 2018). PGD observations are available at near‐field stations prior to rupture completion at roughly the half duration of rupture (Goldberg et al., 2018). Thus, GNSS observations demonstrate consistency with a middle‐of‐the‐road view that there is, at a minimum, weak determinism in the rupture process, meaning that final properties can be inferred sometime between initial observations (~5 s) and rupture completion. The evidence for weak determinism in near‐field GNSS observations suggests that perhaps magnitude saturation is an artifact of observation using high‐pass filtered inertial seismic instrumentation. GNSS unfortunately has lower sensitivity than seismic instrumentation, and typically much lower sampling rates, rendering it insensitive to the small initial ground motions, such as the earliest onset P‐waves.

Seismogeodesy, broadly defined as the optimal combination of collocated seismic and geodetic data, provides a favorable dataset for exploring the subtleties of early observations (Bock et al., 2011; Crowell et al., 2013; Emore et al., 2007; Nikolaidis et al., 2001). The combination dataset has the temporal resolution of the seismic instrumentation, and results in broadband velocity and displacement time series that are more accurate than those derived from integrated strong‐motion accelerations. The seismogeodetic displacement records maintain spectral fidelity at long periods and down to the 0‐Hz static offset and have reduced noise compared to GNSS‐only displacements (Saunders et al., 2016). The seismogeodetic velocities are sensitive enough to detect seismic P‐wave arrivals, allowing proper identification of the earliest earthquake signals (Goldberg & Bock, 2017).

Crowell et al. (2013) studied a small set of seismogeodetic waveforms for P‐wave amplitude magnitude scaling. The study noted that while strong‐motion‐accelerometer‐derived P‐wave amplitude measurements in 3‐ and 5‐s observation windows show no signs of scaling as a function of final magnitude, seismogeodetic P‐wave amplitude seemed to scale for earthquakes as large as M9. They therefore suggested that the initial (3 and 5 s) P‐wave amplitude derived from the unfiltered seismogeodetic displacement record might be indicative of final magnitude, even for large events, further arguing that this surprising result was due to long period signals in the seismogeodetic waveforms, which are absent in the filtered inertial seismic recordings. Seismic and geodetic stations are not usually collocated; thus, this study was limited to a dataset of five globally distributed events, with 118 total recordings including two events with only three observing stations.

**Figure 1:**
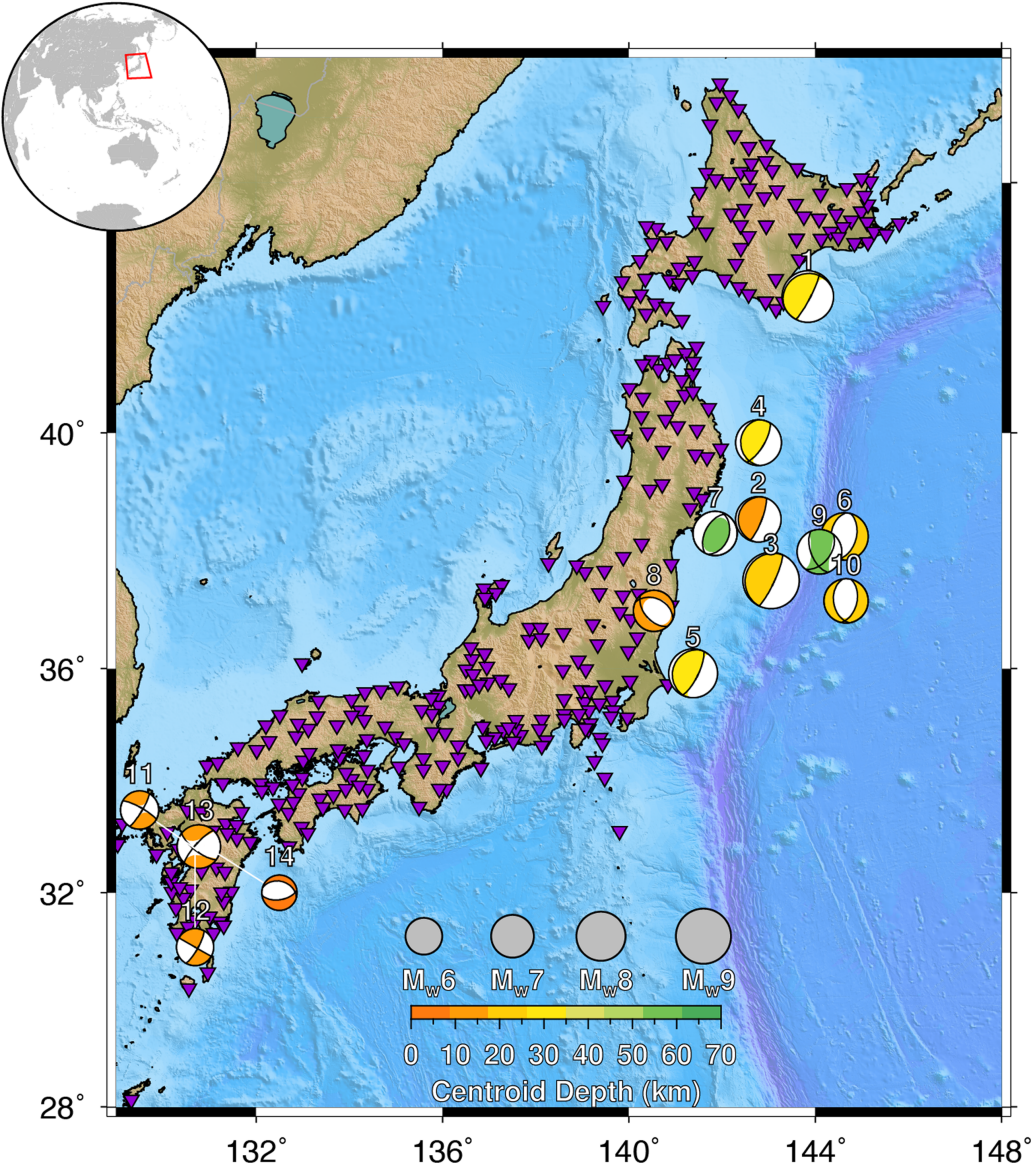
Location of collocated GNSS and strong‐motion accelerometer stations pairs (purple triangles) and earthquakes (colored focal mechanisms) in the analyzed dataset. Circle size corresponds to magnitude and color corresponds to hypocentral depth. The numbers listed above each focal mechanism correspond to the event number in Table 1.

The availability of new seismogeodetic datasets allows us to investigate this P‐wave‐amplitude scaling metric more fully. Here, we present the results from 14 medium‐to‐large magnitude events (M_w_5.7‐9.1) in Japan, each observed by at least 22 collocated GNSS and accelerometer seismic stations (Figure 1, Table 1) for a total of 592 recordings. Our goal is to determine whether the larger seismogeodetic dataset is consistent with P‐wave amplitude scaling up to large magnitudes. We will demonstrate that despite the broadband nature of the seismogeodetic observations, seismogeodetic P‐wave amplitude measurements saturate in a similar manner to inertial seismic recordings and are not diagnostic of final magnitude. We discuss the implications of our results for best practices of ground motion prediction in the context of earthquake early warning.

**Table 1:** Earthquake Event Information for the Fourteen Events Included in the Seismogeodetic Dataset Analysis.

Location	M_w_	Origin time (UTC)	Hypocenter			Number of stations
			Lon (°E)	Lat (°N)	Dep (km)	
Tokachi‐oki^1^	8.3	2003‐09‐25 19:50:07.64	144.0785	41.7797	42.0	22
Miyagi^2^	7.3	2011‐03‐09 02:45:12.97	143.2798	38.3285	8.3	43
Tohoku‐oki^3^	9.1	2011‐03‐11 05:46:18.12	142.3730	38.2970	29.0	54
Iwate^4^	7.4	2011‐03‐11 06:08:53.05	142.7815	39.8390	31.7	24
Ibaraki^5^	7.9	2011‐03‐11 06:15:34.46	141.2653	36.1083	43.2	25
N. Honshu^6^	7.6	2011‐03‐11 06:25:44.04	144.8940	37.8367	34.0	64
Miyagi^7^	7.1	2011‐04‐07 14:32:43.43	141.9237	38.2028	60.7	84
E. Fukushima^8^	6.7	2011‐04‐11 08:16:12.02	140.6727	36.9457	6.4	27
N. Honshu^9^	7.2	2012‐12‐07 08:18:20.28	144.3153	37.8158	46.0	60
N. Honshu^10^	7.1	2013‐10‐25 17:10:18.39	144.5687	37.1963	56.0	25
Kumamoto^11^	6.2	2016‐04‐14 12:26:34.43	130.8087	32.7414	11.4	43
Kumamoto^12^	6.0	2016‐04‐14 15:03:46.45	130.7777	32.7007	6.7	34
Kumamoto^13^	7.0	2016‐04‐15 16:25:05.47	130.7630	32.7545	12.5	59
Kumamoto^14^	5.7	2016‐04‐15 16:45:55.45	130.8990	32.8632	10.6	28

*Note*. Earthquake origin time and hypocenter location are from NIED (http://www.fnet.bosai.go.jp). Origin time is given as year‐month‐day hour:minute:second. Magnitude is from Global CMT (http://globalCMT.org).

## 2 Dataset

We evaluate the deterministic nature of seismogeodetically derived P‐wave amplitude using the dense accelerometer and GNSS networks available in Japan. The strong‐motion acceleration data are available from the National Research Institute for Earth Science and Disaster Resilience (NIED, http://www.kyoshin.bosai.go.jp) KiK‐net and K‐NET datasets. GNSS data were retrieved from the Geospatial Information Authority (http://www.gsi.go.jp), which operates the GNSS Earth Observation Network System (GEONET). We consider strong‐motion accelerometer and GNSS station pairs to be collocated if they are within 2 km of one another. In Japan, more than 300 such station pairs exist. This inter‐instrument distance is a conservative criterion based upon previous work demonstrating agreement between instrument pairs with up to 4 km separation (Emore et al., 2007).

The high‐rate (1 Hz) GEONET observations were processed using precise point positioning (PPP; Geng et al., 2013; Zumberge et al., 1997) to produce displacements in a local north, east, up reference frame for each earthquake. Although we will use the term “GNSS” to describe the GEONET observations, note that our processing ingests only the Global Positioning System (GPS) observations to derive the displacement time series. The GNSS displacements were then merged with the strong‐motion accelerations from collocated KiK‐net/K‐NET sites using the Kalman filter method (Bock et al., 2011) to create the seismogeodetic time series. The hypocentral distance, *R*, of each source‐station pair was computed from the hypocenter location determined by NIED (http://www.fnet.bosai.go.jp). The P‐wave arrival times were selected manually from the vertical acceleration waveforms at each site. The accelerometer stations are operated in triggered‐mode, sometimes resulting in late triggers such that the records at some stations begin after the first P‐wave arrivals. If a record appears to begin after the P‐wave arrival, the station pair is eliminated from the analysis. As a result, the number of collocated stations used to study each earthquake is reduced to between 22 and 84 sites per event (Table 1).

We consider 14 earthquakes recorded on collocated seismogeodetic stations, occurring between 2003 and 2016, with magnitudes of M_w_5.7 to M_w_9.1 (Figure 1; Table 1). These same events were previously used to investigate the seismic moment evolution using GNSS displacements alone (Goldberg et al., 2018), and the processed strong motion acceleration and GNSS displacement waveforms are freely available online as detailed in Ruhl et al. (2018, 2019).

## 3 Methods

We consider observation periods of 3 and 5 s after P‐wave arrival time following Crowell et al. (2013), and evaluate peak horizontal amplitude, *P*_*h*_, peak vertical amplitude, *P*_*v*_, and peak amplitude of the three‐component sum of squares, *P*_3_, where

(1) $$\begin{array}{l} P_{h}=\max\left(\sqrt{N{\left(t\right)}^{2}+E{\left(t\right)}^{2}}\right),\\ P_{v}=\max\left(\sqrt{U{\left(t\right)}^{2}}\right),\\ P_{3}=\max\left(\sqrt{N{\left(t\right)}^{2}+E{\left(t\right)}^{2}+U}{\left(t\right)}^{2}\right), \end{array}$$

and *N(t)*, *E(t)*, and *U(t)* are the north‐south, east‐west, and up‐down component displacement waveforms, respectively. An example of the maximum P‐wave amplitude selection for two example station pairs during the 2011 M_w_9.1 Tohoku‐oki mainshock can be found in Figure S1. We present the results for *P*_V_ and a 3‐s observation period in the main text, with remaining results available in the Supporting Information. A fixed time window is important for potential operational utilities (i.e., earthquake or tsunami early warning), however we note that this choice results in sampling a different portion of the source process for earthquakes of different magnitude. For example, a fixed window of 5 s may sample the full duration of a M_w_6 event, but only the initial moment release of an M_w_7 (rupture duration ~30 s).

To investigate whether magnitude saturation is due to the loss of the long‐period band as a result of typical high‐pass filtering practices of integrated inertial seismic observations, we compare the broadband, unfiltered seismogeodetic combination to the same waveforms filtered with a corner period of 13 s, as is typical of seismic‐only analyses (e.g. Trugman et al., 2019; Wu & Zhao, 2006).

We use the magnitude scaling law functional form proposed by Wu and Zhao (2006), equation (2), which relates peak P‐wave amplitude, *P*_*d*_, to moment magnitude, M_w_, and hypocentral distance, *R*, assuming that P‐wave amplitude attenuates linearly on a log‐log scale.

(2) $$\log_{10}P_{d}=A+B\cdot M_{w}+C\cdot \log_{10}R.$$

We compare the observed amplitudes to those predicted by the functional form in equation (2), using coefficients (*A*, *B*, *C*) determined by previous studies (Kuyuk & Allen, 2013; Tsang et al., 2007; Wu & Zhao, 2006; see Table S1). We use hypocentral distance, rather than more commonly used distance metrics for ground motion prediction such as the finite‐fault distance or Joyner‐Boore distance (e.g., Boore et al., 1997) because hypocentral distance is available under the time constraint of EEW. We evaluate if P‐wave amplitudes measured with seismogeodesy are sufficient for estimating the final magnitude of large earthquakes, or rather if P‐wave amplitude measurements saturate for large magnitude events despite the broadband nature of the seismogeodetic combination.

## 4 Results

**Figure 2:**
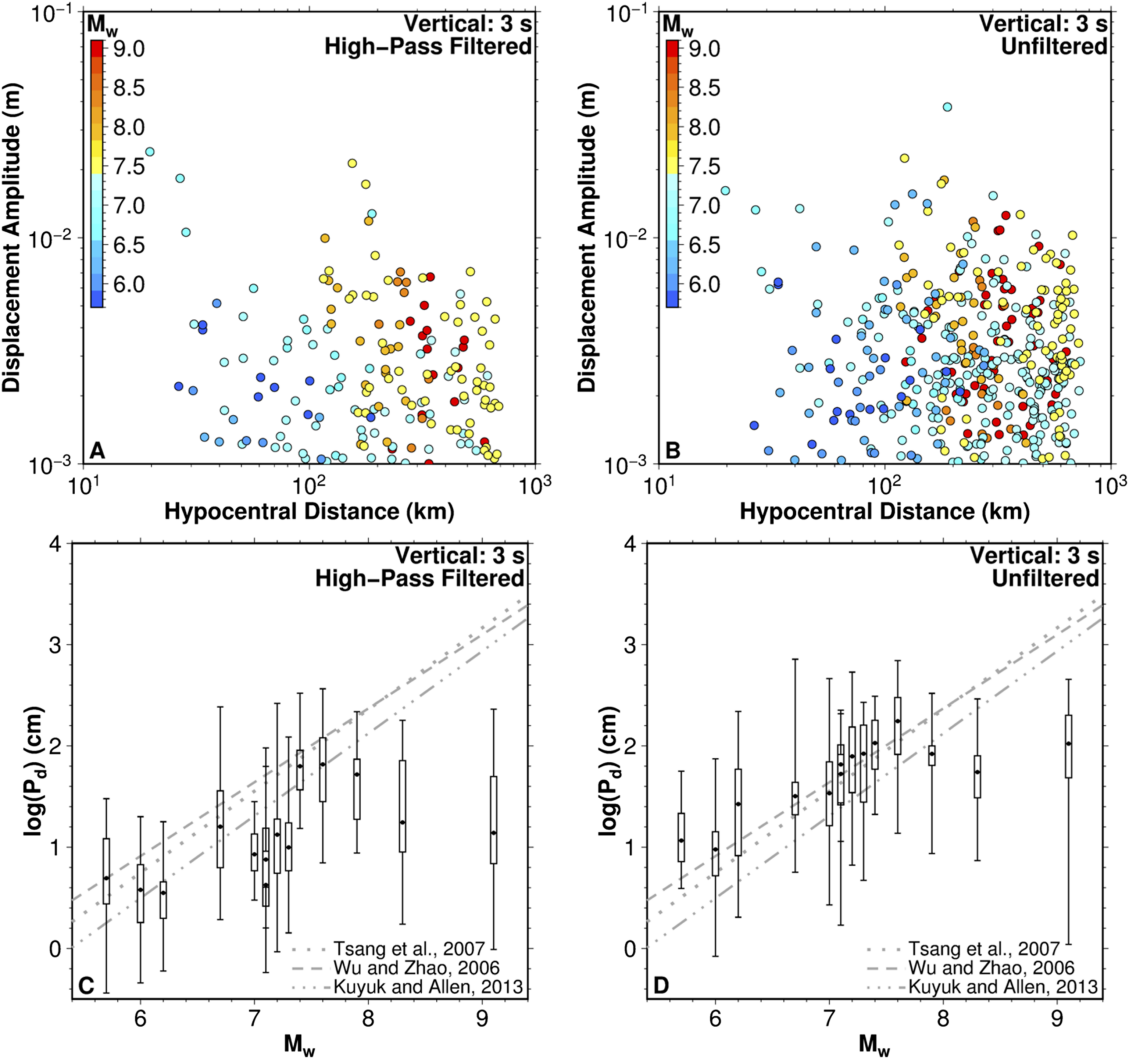
High‐pass filtered (left) and unfiltered (right) vertical component displacement amplitudes with 3‐s observation window after P‐wave arrival. (a,b) Maximum P‐wave displacement amplitude (in meters) as a function of hypocentral distance. (c,d) The base 10 logarithm of P‐wave amplitudes (in centimeters) corrected to a hypocentral distance of 1 km as a function of earthquake magnitude. Each box and whisker represents a single earthquake from Table 1. Dashed lines show the relations derived in previous studies (see Table S1). The high‐pass filtered dataset (a,c) is an approximation of the real‐time seismic methodology, while the unfiltered dataset (b,d) represents the optimal broadband seismogeodetic approach.

Visually, there is no discernible difference between the maximum P‐wave amplitude of events of significantly different magnitudes (M7.5–M9) measured in either the first 3 or 5 s after the P‐wave arrival for any component considered, whether filtered or unfiltered (Figures 2a and 2b; see Figures S2 to S6 for results of 5‐s *P*_*v*_, 3‐s *P*_*h*_, 5‐s *P*_*h*_, 3‐s *P*_*3*_, and 5‐s *P*_*3*_, respectively). The observations of earthquakes of large magnitude overlap, and no clear pattern is visible. For example, the observations for the M9 Tohoku‐oki earthquake overlap with earthquakes as small as M7.5. This result is in contrast with the previous study by Crowell et al. (2013), which observed separation between a smaller seismogeodetic dataset of globally distributed earthquakes, yet consistent with previous analysis showing that the M9 Tohoku‐oki event had a slow initial moment release (e.g., Noda & Ellsworth, 2017; Trugman et al., 2019).

We correct the P‐wave amplitude observations for 1/R geometric spreading and reference them to a common hypocentral distance, using a reference distance of 1 km to show the distribution of amplitudes within each earthquake in the database (Figures 2c and 2d). We compare results for the seismogeodetic time series, high‐pass filtered with a corner period of 13 s (Figure 2c), which imitate seismic analyses (e.g., Wu & Zhao, 2006), to results using unfiltered seismogeodetic waveforms (Figure 2d). Our analysis shows that indeed, as was proposed by Crowell et al. (2013), the high‐pass filtering of the displacement waveforms can introduce a significant reduction of the observed maximum amplitudes. Further, we see that this effect is greater for the larger magnitude earthquakes, where unfiltered displacement amplitudes are larger. However, in spite of this more complete and broadband measurement of the P‐wave amplitudes from the unfiltered seismogeodetic dataset, we observe the same pattern in comparison to the magnitude scaling relations. The observed P‐wave amplitudes are increasingly overestimated by the scaling relations as the magnitude of the earthquake in question increases. This behavior in both filtered and unfiltered recordings is consistent with saturation in the scaling properties of P‐wave amplitude for large magnitude earthquakes, observed to occur near M_w_7.5. In a real‐time EEW context, for example, using the observed log (*P*_*d*_) values to estimate M_w_ would be poorly constrained given the overlap of log (*P*_*d*_) values across many magnitudes (Figures 2c and 2d). This interpretation is the same for each proposed definition of P‐wave amplitude, regardless of component of motion or search window (3 or 5 s) considered (see Figures S2 to S6).

## 5 Discussion

In the present study, the larger events (>M7.5) appear indistinguishable this early (i.e., 3–5 s) in the rupture process, which is consistent with the magnitude saturation typical of seismic‐only observations and previous work describing the expected behavior of P‐wave amplitudes at these observation windows (Trugman et al., 2019). While the P‐wave amplitude measurements are generally larger in the unfiltered dataset, the evidence for magnitude saturation remains. The results of this study are in contrast with those of Crowell et al. (2013), which suggested that maximum P‐wave amplitude observed in the first 3–5 s after initial earthquake observation, scales with magnitude when measured from the seismogeodetic dataset even for large magnitude events. The Crowell et al. (2013) study was completed with a significantly smaller and globally distributed dataset due to the limited availability of historical seismogeodetic datasets at the time, and therefore we consider the results of this larger, regionally limited study to be more robust. We find no evidence for strong determinism in the P‐wave amplitude of large magnitude earthquakes, consistent with more recent seismic‐only analyses (e.g., Meier et al., 2016). Rather, we find that magnitude saturation from P‐wave characteristics is inherent to large earthquake dynamics and not due to instrumental limitations. Our results suggest that at present, the observation of PGD at near‐field GNSS sites (Goldberg et al., 2018) is the earliest real‐time metric for magnitude scaling from direct displacement observations, requiring, for example, ~35 s of observation at stations within 90 km of a M_w_8.5 event. This view is supported by studies of the source time functions of large earthquakes which see similar *weakly* deterministic behavior, characterized by the ability to determine magnitude at some point after the first few seconds, but prior to completion of rupture (Meier et al., 2017; Melgar & Hayes, 2017).

Our findings have important implications for the timeliness of operational earthquake early warning products because magnitude is a key parameter for predicting ground motions. The lack of strong determinism imposes a limitation on the reliability of near‐source ground motion predictions because the proper size of large magnitude earthquakes may not be resolved for many tens of seconds after initial observation. Operationally, ground motion warnings should identify the expected intensity of shaking, such as the Modified Mercalli intensity (MMI) thresholds. The subject of MMI‐style warning times derived from earthquake source estimates has been previously investigated assuming the case where final earthquake properties are known instantaneously (Minson et al., 2019) and the weakly deterministic case in which magnitude can be evaluated at roughly the rupture half duration (Minson et al., 2018). In demonstrating the lack of strong determinism in broadband seismogeodetic P‐wave amplitude observations, we suggest that the timeliness of EEW evaluated in Minson et al. (2018), which assumes that source size estimates grow with moment release, is most realistic. That study found that earthquake‐source‐based ground‐motion predictions are most useful for users tolerant of false alerts and willing to choose lower warning thresholds in order to allow longer warning times for felt events. Similarly, Ruhl et al. (2019) describe the improvement to the timeliness and accuracy of EEW MMI‐threshold estimates for large magnitude events (>M7.5) upon inclusion of geodetic constraints, increasing user warning times and improving the cost‐savings performance, especially for users averse to false alerts. In short, even if only partial knowledge of the earthquake source process is available, this can still be used to warn in advance of strong shaking arriving at a particular site.

Although we have not found evidence for strong determinism for large events using early onset P‐wave metrics, we note that ongoing work into the manifestation of weak determinism has potential to further improve the timeliness of an alert. For example, teleseismic observations have been used to demonstrate that seismic moment rate is indicative of magnitude within the first tens of seconds (Melgar & Hayes, 2019). Danré et al. (2019) suggest from an analysis of the roughness of source time functions that perhaps still shorter timescales are possible. Further investigation of such findings in the near‐field may elucidate observational qualities somewhere between initial P‐wave and peak ground displacement metrics to allow faster estimates of final properties.

Finally, we acknowledge that availability of observational data is still a major limitation for the study of early rupture properties in the near‐field of large earthquakes. Despite the large and overlapping seismic and GNSS datasets in Japan, the number of collocated stations that properly recorded the earliest seismic arrivals is relatively small. Seismogeodetic data availability is more problematic elsewhere where collocations are far more rare. Furthermore, there are very few large (>M8) earthquakes that have been recorded with collocated seismic and GNSS instrumentation. Presently, the M_w_9.1 Tohoku‐oki earthquake is the only well‐instrumented event of great magnitude, and therefore studies such as this one assume that potential future great megathrust events share similar properties to that earthquake. However, the M9.2 2004 Sumatra earthquake (observed with mainly teleseismic data) began markedly differently, with slow rupture and little slip for nearly a minute prior to ramping up to become a great, long‐duration earthquake (Ammon et al., 2005). Moreover, our dataset includes mostly offshore events, thus our results may not be broadly applicable to other tectonic regions, such as damaging crustal events in California, USA. While our results echo those of Meier et al. (2016) and show no evidence of strong determinism, it is unlikely that this topic will be fully resolved without additional near‐field observational data from future large earthquakes.

## 6 Conclusions

Previous study of a limited, global dataset suggested that magnitude saturation is not inherent to large earthquake dynamics, but rather is due to unreliable observation of long‐period signals using inertial seismic instrumentation. Seismogeodetic methods allow full spectral fidelity in the observation of large magnitude events, providing a broadband dataset with which to evaluate whether P‐wave amplitude is indicative of final earthquake magnitude. Using a larger dataset of medium‐to‐great earthquakes, and limiting our study area to reduce regional effects, we have shown that seismogeodetically derived P‐wave amplitude is not a reliable predictor of magnitude, opposing the notion of strong determinism in the first few seconds of rupture. Instead, we find that magnitude saturation from P‐wave metrics measured in the first few seconds is inherent to large earthquake dynamics, and is not a consequence of instrumental limitations.

## Supporting information

Supporting Information S1
