# Supplementary material for: Seismogeodetic P‐wave Amplitude: No Evidence for Strong Determinism
Source: Geophys Res Lett. 2019 Oct 29;46(20):11118–26. doi: 10.1029/2019GL083624 (PMC6919942; doi:10.1029/2019GL083624)
Supplement: Supplementary file 1 — Supporting Information S1 [file GRL-46-11118-s001.docx]

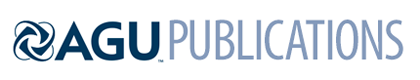


*Geophysical Research Letters*

Supporting Information for

**Seismogeodetic P-wave Amplitude: No Evidence for Strong Determinism**

D.E. Goldberg^1,2^, D. Melgar^2^, and Y. Bock^1^

^1^Institute of Geophysics and Planetary Physics, Scripps Institution of Oceanography, University of California San Diego, La Jolla, California

^2^Department of Earth Sciences, University of Oregon, Eugene, Oregon

**Contents of this file**

Figures S1 to S6

Table S1

**Introduction**

This supporting information describes the selection of P-wave amplitude from the seismogeodetic time series. We include the results of P-wave amplitude scaling for all remaining time windows (3 and 5 seconds) and components of motion (three-component, vertical, horizontal) considered. We include a table of the coefficients of the magnitude scaling relations proposed in previous works used as a reference in this study.


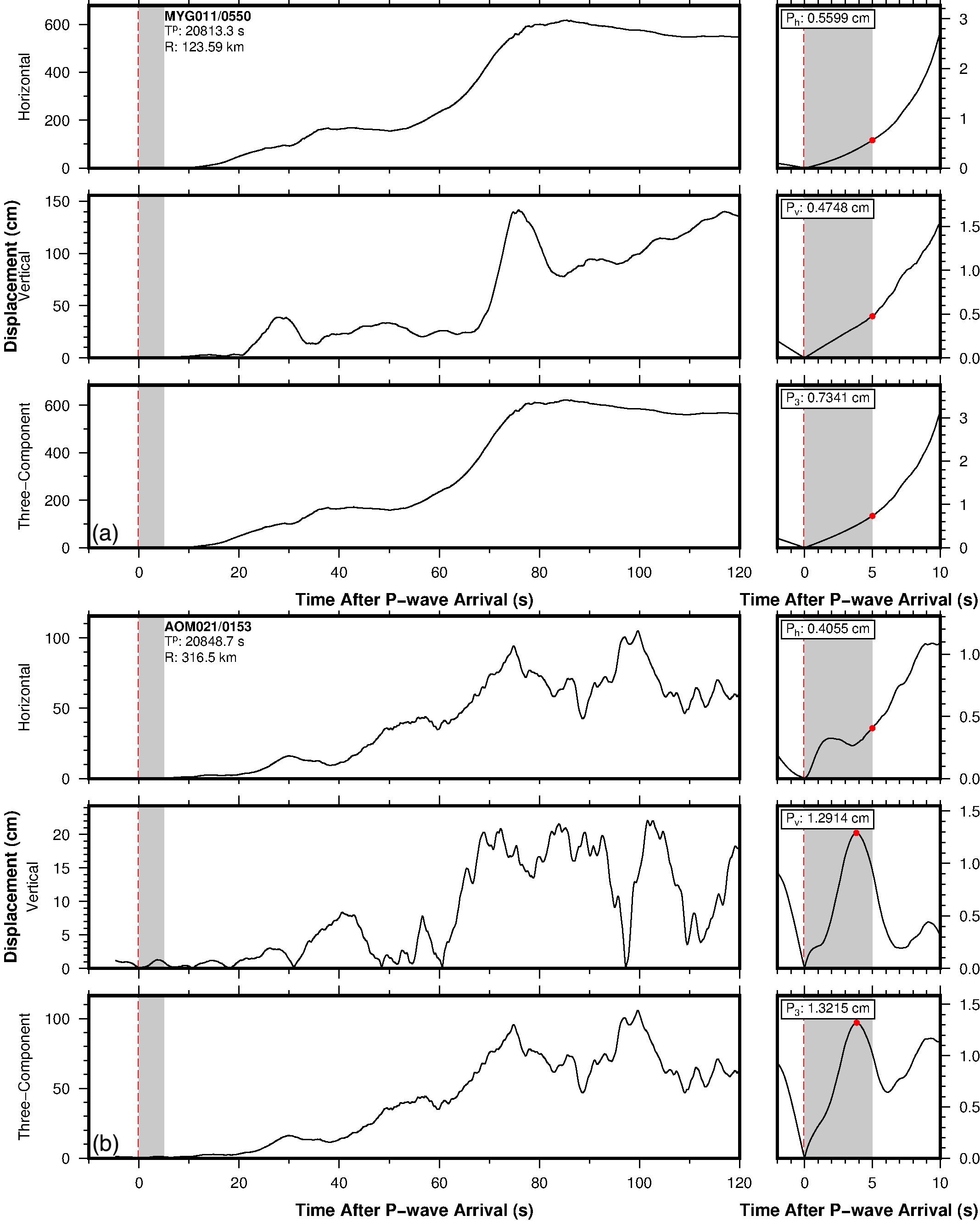


**Figure S1**. Example of maximum P-wave amplitude selection for a 5-second search window for (a) station pair MYG011/0550 and (b) station pair AOM021/0153 during the 2011 M_w_9.1 Tohoku-oki earthquake. Left: Time series of horizontal (top), vertical (middle) and three-component (bottom) seismogeodetic displacement. Red dashed line denotes P-wave arrival time at the station. The grey shaded region is the allowed search window, shown here for 5 seconds after the P-wave arrival. *T^P^* is the P-wave arrival time in seconds after start of day and *R* is the hypocentral distance. Right: Detailed view of the first 10 seconds of the time series (same component as left), showing the P-wave amplitude selection. *P_h_*, *P_v_*, and *P_3_* are the horizontal, vertical, and three-component sum of squares amplitudes, respectively. The red dot denotes the maximum displacement amplitude within the shaded region.


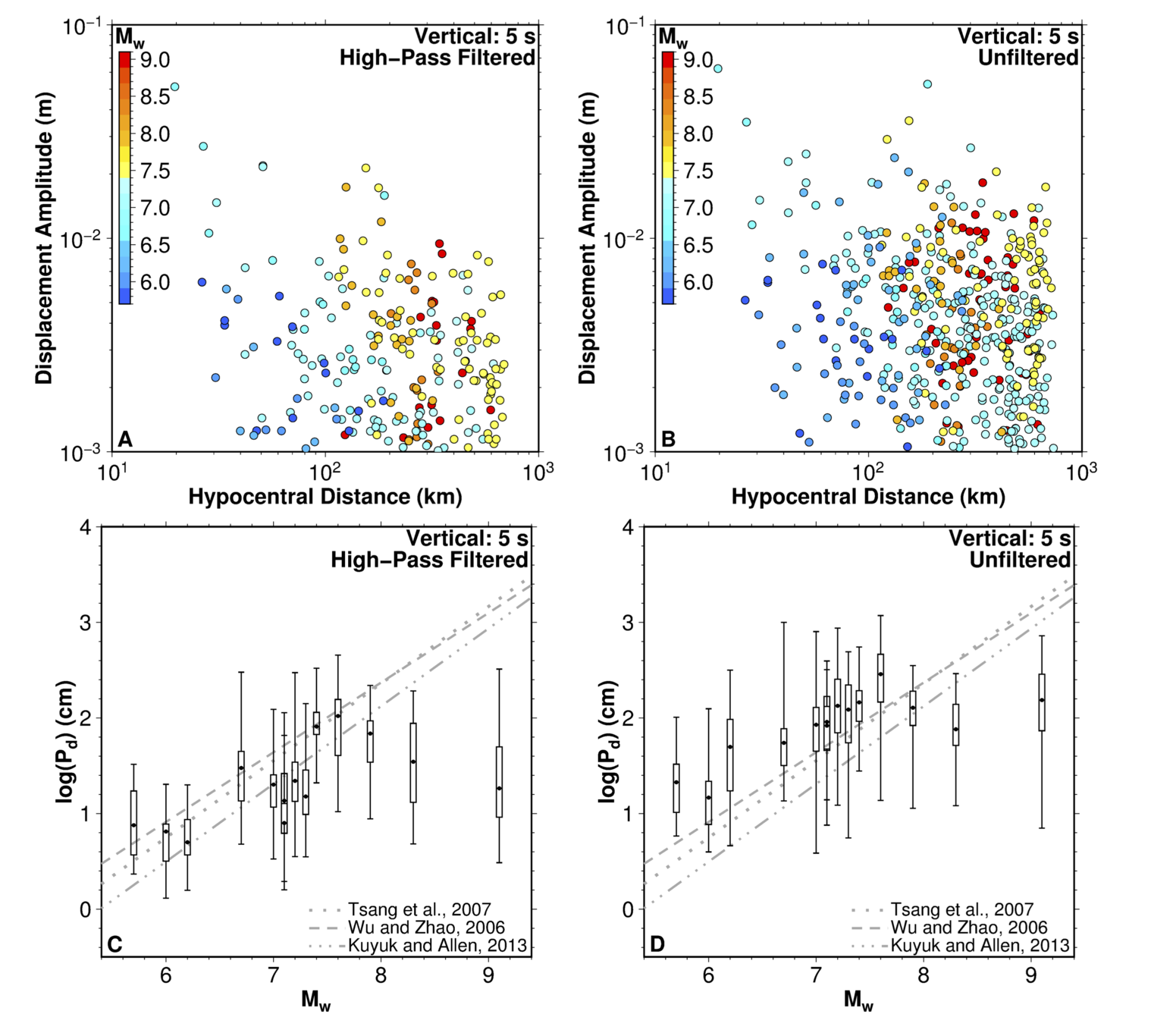


**Figure S2**. High-pass filtered (left) and unfiltered (right) vertical component displacement results with 5-second observation window after P-wave arrival. (A,B) Maximum P-wave displacement amplitude (in meters) as a function of hypocentral distance. (C,D) The base 10 logarithm of P-wave amplitudes (in centimeters) corrected to a hypocentral distance of 1 km as a function of earthquake magnitude. Each box and whisker represents a single earthquake from Table 1. Dashed lines show the relations derived in previous studies (see Table S1). The high-pass filtered dataset (A,C) is an approximation of the real-time seismic methodology, while the unfiltered dataset (B,D) represents the optimal broadband seismogeodetic approach.


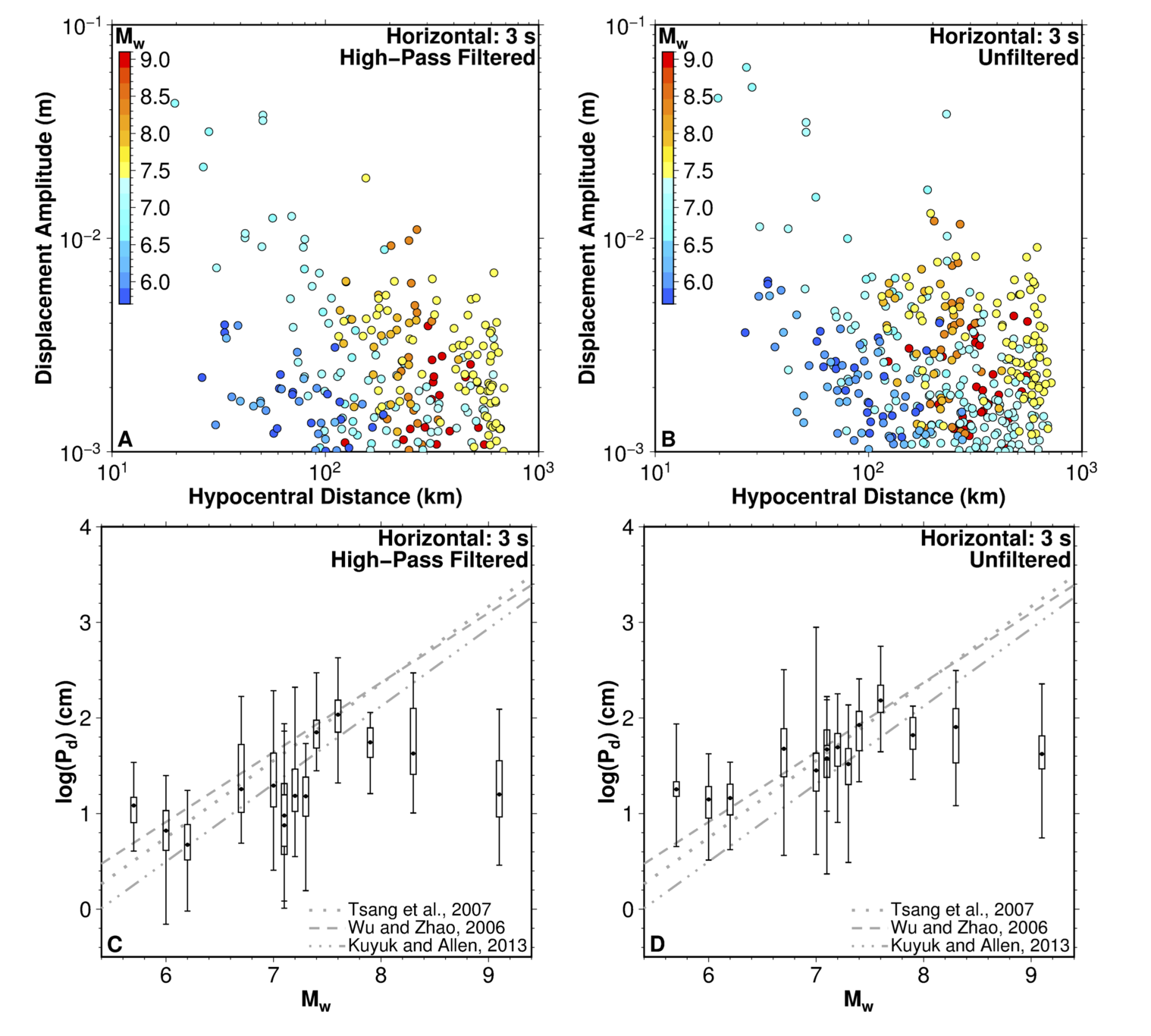


**Figure S3**. High-pass filtered (left) and unfiltered (right) horizontal displacement results with 3-second observation window after P-wave arrival. (A,B) Maximum P-wave displacement amplitude (in meters) as a function of hypocentral distance. (C,D) The base 10 logarithm of P-wave amplitudes (in centimeters) corrected to a hypocentral distance of 1 km as a function of earthquake magnitude. Each box and whisker represents a single earthquake from Table 1. Dashed lines show the relations derived in previous studies (see Table S1). The high-pass filtered dataset (A,C) is an approximation of the real-time seismic methodology, while the unfiltered dataset (B,D) represents the optimal broadband seismogeodetic approach.


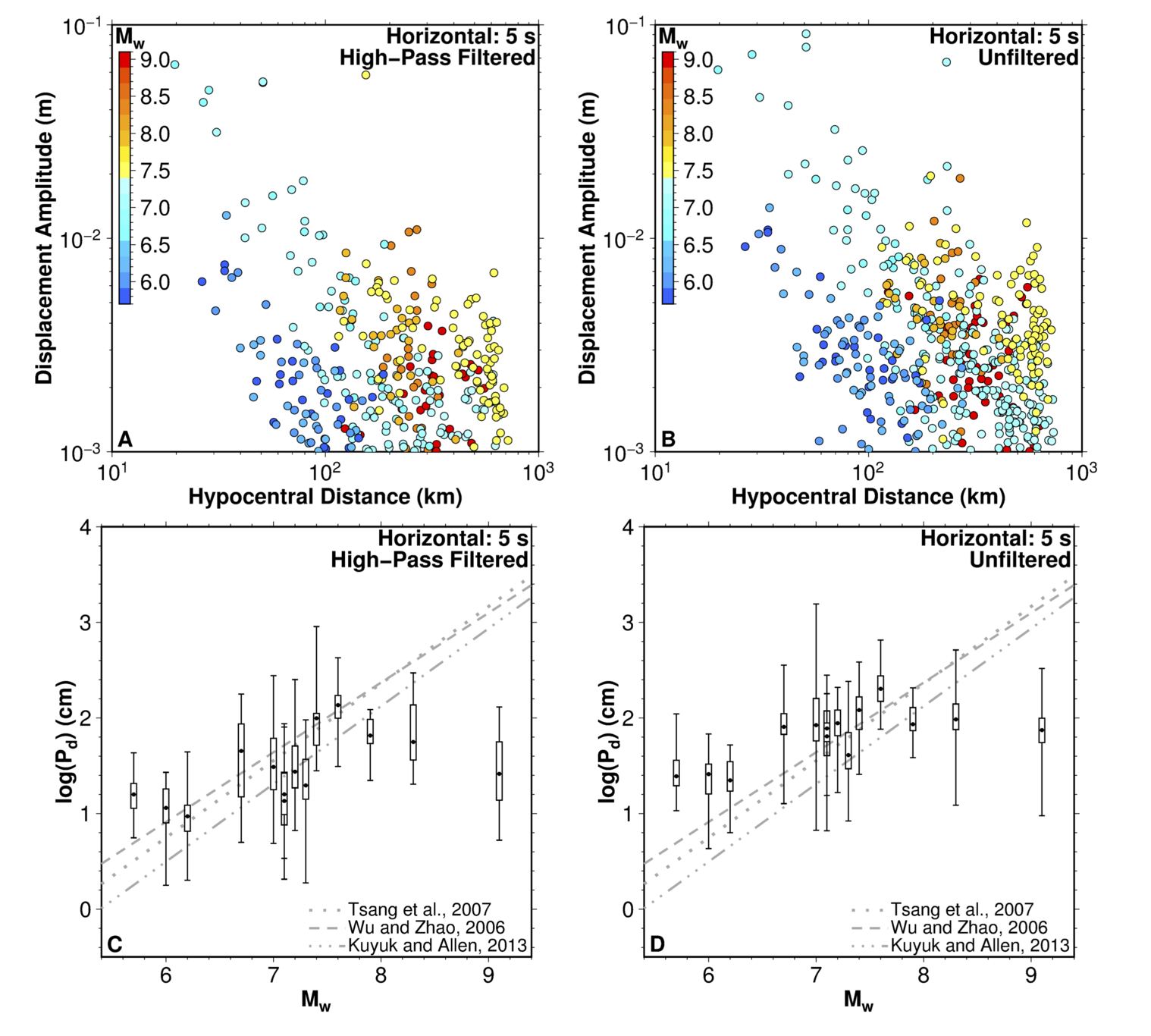


**Figure S4**. High-pass filtered (left) and unfiltered (right) horizontal displacement results with 5-second observation window after P-wave arrival. (A,B) Maximum P-wave displacement amplitude (in meters) as a function of hypocentral distance. (C,D) The base 10 logarithm of P-wave amplitudes (in centimeters) corrected to a hypocentral distance of 1 km as a function of earthquake magnitude. Each box and whisker represents a single earthquake from Table 1. Dashed lines show the relations derived in previous studies (see Table S1). The high-pass filtered dataset (A,C) is an approximation of the real-time seismic methodology, while the unfiltered dataset (B,D) represents the optimal broadband seismogeodetic approach.


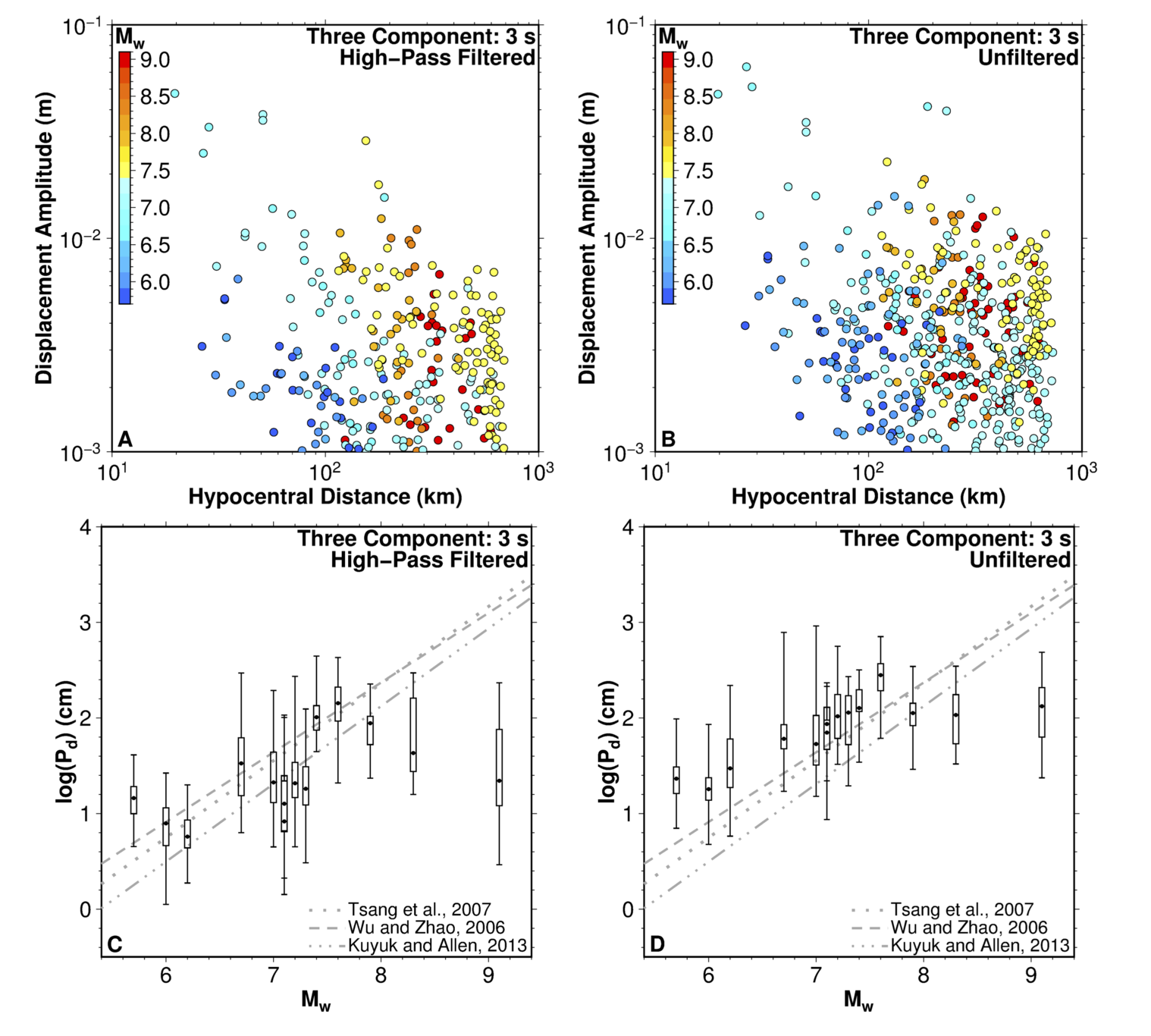


**Figure S5**. High-pass filtered (left) and unfiltered (right) three component displacement results with 3-second observation window after P-wave arrival. (A,B) Maximum P-wave displacement amplitude (in meters) as a function of hypocentral distance. (C,D) The base 10 logarithm of P-wave amplitudes (in centimeters) corrected to a hypocentral distance of 1 km as a function of earthquake magnitude. Each box and whisker represents a single earthquake from Table 1. Dashed lines show the relations derived in previous studies (see Table S1). The high-pass filtered dataset (A,C) is an approximation of the real-time seismic methodology, while the unfiltered dataset (B,D) represents the optimal broadband seismogeodetic approach.


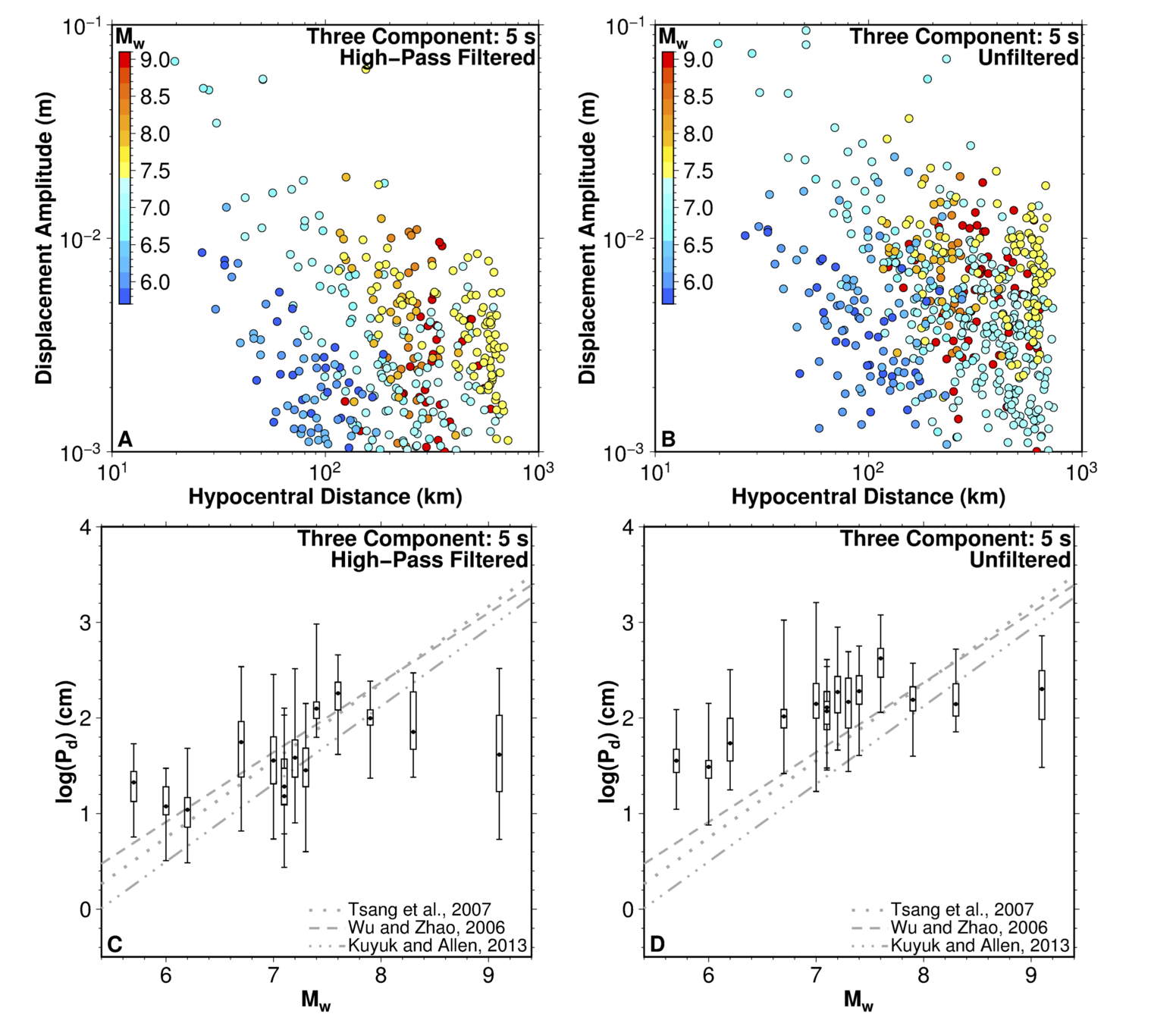


**Figure S6**. High-pass filtered (left) and unfiltered (right) three component displacement results with 5-second observation window after P-wave arrival. (A,B) Maximum P-wave displacement amplitude (in meters) as a function of hypocentral distance. (C,D) The base 10 logarithm of P-wave amplitudes (in centimeters) corrected to a hypocentral distance of 1 km as a function of earthquake magnitude. Each box and whisker represents a single earthquake from Table 1. Dashed lines show the relations derived in previous studies (see Table S1). The high-pass filtered dataset (A,C) is an approximation of the real-time seismic methodology, while the unfiltered dataset (B,D) represents the optimal broadband seismogeodetic approach.

**Table S1.** P-wave magnitude scaling relation coefficients from previous studies.

| ${log}_{10}P_{d} =A+B\cdot M_{w}+C\cdot{log}_{10}R$ | | | |
| --- | --- | --- | --- |
| **Previous Study** | ***A*** | ***B*** | ***C*** |
| **Tsang et al., 2007** | -4.089 | 0.806 | -1.331 |
| **Wu & Zhao, 2006** | -3.463 | 0.729 | -1.337 |
| **Kuyuk & Allen, 2013** | -4.382 | 0.813 | -1.122 |
